# New pharmacological therapies in pure OCD: about a case

**DOI:** 10.1192/j.eurpsy.2025.1712

**Published:** 2025-08-26

**Authors:** C. Garcia Bernal

**Affiliations:** Psychiatry, Private Medical Center Dra. Cristina Garcia Bernal, Seville, Spain

## Abstract

**Introduction:**

Obsessive disorders occur in 2 to 3% of the world’s population. They are very often underdiagnosed, causing great discomfort to the person.

**Objectives:**

With this clinical case I would like us to be able to use new therapeutic strategies that, even though they are outside the technical specifications, work and improve the quality of life of patients with obsessive disorder.

**Methods:**

I present the clinical case of a 14-year-old male who consulted for obsessive symptoms a year and a half ago.

The patient reports that he cannot stop thinking throughout the day, any type of absurd thought comes to him and he is not able to stop it, it limits his functionality to the point that a significant academic decline had occurred when previously the minor scored very good grades. He has had angry outbursts at school due to the discomfort his thoughts cause him. I treated with Clomipramine 75 mg and Clonazepam solution as a rescue anxiolytic and appointment in 3 weeks.

**Results:**

The patient progressively improves in the organization of thought and decreases obsessive content, increasing the medication with Clomipramine up to 150 mg at night. After two months, it is the mother who consults because she sees her son very tired, he had gained a lot of weight and was complaining of dry mouth (side effects) and it continues to persist and worsen functionality. Given the patient’s lack of complete improvement and the obvious side effects, I decided to first progressively change Clomipramine to Vortioxetine up to 40 mg. Upon evaluation, the patient feels better about the obsessive content and no side effects appear, but cognitive rigidity, inflexibility, and functional and academic decline persist. That is why two months later, I started Cariprazine 1.5 mg and in a few days the patient felt more animated, eager to do things and concentrated better, even so I decided to go up to 3 mg and that is when the patient reported a notable improvement, also reported by his mother and his psychologist. He is currently continuing with this treatment.

**Conclusions:**

Neither Vortioxetine nor Cariprazine has an indication in the technical specifications for obsessive disorders, but there are publications that show that it works without having the side effects of drugs with a dirtier pharmacological profile.

**Disclosure of Interest:**

None Declared

